# Evaluation of Ecotoxicological Risks Related to the Discharge of Combined Sewer Overflows (CSOs) in a Periurban River 

**DOI:** 10.3390/ijerph10072670

**Published:** 2013-06-28

**Authors:** Ruth Angerville, Yves Perrodin, Christine Bazin, Evens Emmanuel

**Affiliations:** 1 Université de Lyon, ENTPE, UMR CNRS 5023 LEHNA, 2 rue Maurice Audin, Vaulx-en-Velin 69518, France; E-Mail: angerville_ruth@yahoo.com; 2 Université Quisqueya, Laboratoire LAQUE, BP 796, Port-au-Prince, Haïti; E-Mail: evens.emmanuel@gmail.com; 3 INSAVALOR, POLDEN, 20 avenue Albert Einstein, Villeurbanne Cedex 69621, France; E-Mail: christine.bazin@insavalor.fr

**Keywords:** Combined Sewer Overflows, ecotoxicological risk assessment, environmental management, river, bioassay

## Abstract

Discharges of Combined Sewer Overflows (CSOs) into periurban rivers present risks for the concerned aquatic ecosystems. In this work, a specific ecotoxicological risk assessment methodology has been developed as management tool to municipalities equipped with CSOs. This methodology comprises a detailed description of the spatio-temporal system involved, the choice of ecological targets to be preserved, and carrying out bioassays adapted to each compartment of the river receiving CSOs. Once formulated, this methodology was applied to a river flowing through the outskirts of the city of Lyon in France. The results obtained for the scenario studied showed a moderate risk for organisms of the water column and a major risk for organisms of the benthic and hyporheic zones of the river. The methodology enabled identifying the critical points of the spatio-temporal systems studied, and then making proposals for improving the management of CSOs.

## 1. Introduction

The general trend of urban sprawl generates increasing flows of pollutants discharged into the aquatic environment via urban sewage systems, which include individual wastewaters and rainwater management systems [1,2,3,4]. The sources of the pollutants discharged by the latter are many: wastewater pollutants, atmospheric pollutants, leached pollutants and particles washed away after accumulation on urban surfaces during dry periods, the erosion of urban materials, the return to suspension of sediments accumulated in sewer networks [5,6,7]. This mixture of urban waters discharged into aquatic environments during wet periods, called Combined Sewer Overflow (CSOs), has been the subject of a large number of studies in recent years [8,9,10,11]. The input of pollutants linked to these effluents in the natural environment has numerous consequences, such as increased turbidity due to suspended matter, deoxygenation of the environment due to the input of degradable organic matter and nitrous materials, and toxic effects on aquatic organisms via the input of chemical substances [9,12,13,14,15].

Up to now, no structured methodology for the predictive assessment of ecotoxicological risks related to these specific discharges has been proposed. The deployment of such a methodology is nonetheless vital when designing new rainwater drainage networks, in order to set up adapted management systems if a risk exists [16]. After recalling the fundamentals of ecological risk assessment at international level, this article focuses on the formulation of a risk assessment methodology specific to CSOs, and on its application to a discharge of CSOs located in the periphery of the city of Lyon in France. Finally, improvements to the methodology are proposed for optimising the assessment of risks incurred by host aquatic environments.

## 2. Methodological Approach for the Ecological Risk Assessment

The first Ecological Risk Assessment (ERA) methodologies emerged at the beginning of the 1990s with dawning awareness of the risks liable to impact ecosystems when they are exposed to substances of anthropic origin. In 1992, the United States EPA proposed a framework for the ecological risk assessment of contaminated industrial sites [17]. Following a certain number of works, especially those of Suter [18], this guide was improved to become “The Guidelines for Ecological Risk Assessment” [19] which has now become the reference regarding ERA [16,20]. Since then, this guide has been revised by many countries and adapted for the management of their polluted sites [21,22,23].

In addition, methodologies have been formulated to evaluate ecological risks linked to other problems. Mention can be made of the methodology drawn up by the European Union to evaluate risks relating to chemical substances placed on the market [22,24], and French works on the assessment of ecotoxicological risks linked to dumping continental dredged sediments [25], on the assessment of the ecocompatibility of using wastes [26,27], and on the assessment of ecotoxicological risks linked to hospital effluents [28,29].

Most ERA methods formulated at international level are implemented with four main phases: (1) the formulation of the problem, (2) the characterisation of exposures, (3) the characterisation of effects, and lastly, and (4) the characterisation of the risk itself.

## 3. Formulation of the Risk Assessment Methodology Dedicated to CSOs

### 3.1. Formulation of the Problem

The problem formulation phase comprises notably the description of the studied scenario, the definition of priority objectives, and the formulation of the conceptual model [16,19,20].

#### 3.1.1. Description of the Scenario (Figure 1)

The aim of describing the scenario is to provide a detailed account of the different elements making up the spatio-temporal systems studied. They are related to three major components of the scenario: (i) the source of pollution concerned, (ii) the target ecosystems to be protected, and (iii) the level of exposure of the target ecosystems. 

**Figure 1:**
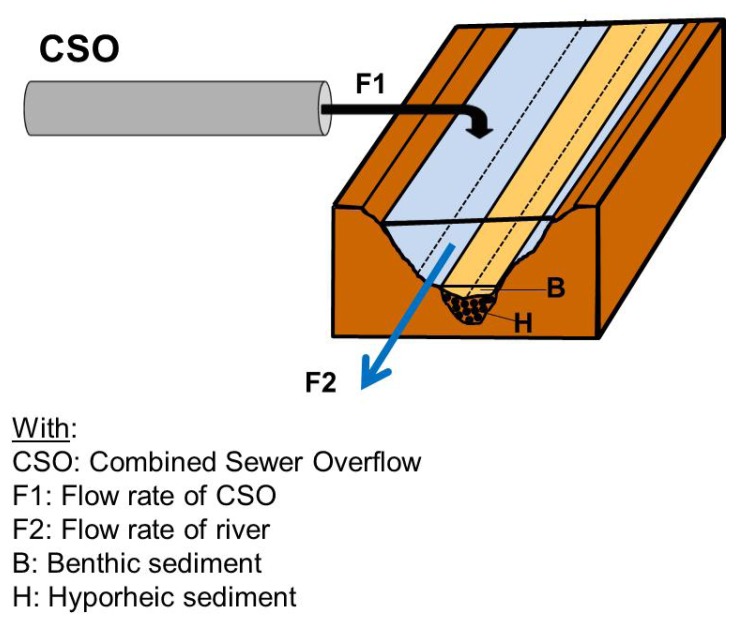
Representation of the scenario studied.

In this case the sources of pollution correspond to CSOs discharged by a storm overflow for which it is necessary to determine the level of concentration in pollutants at the time of the storm. The target organisms to be protected are those of the river receiving the effluent. Three types of organisms can be distinguished in the river, according to the type of substrate they occupy:

- pelagic organisms living in the water column,
- benthic organisms living in the sediments of the river,
- hyporheic organisms living in the sediments situated under the river.

For the organisms of the water column, the trophic levels and potential targets to be considered are the primary producers (including single-cell and multi-cellular green algae), primary consumers (pelagic micro and macro-invertebrates, especially micro-crustaceans), and secondary consumers, including fish (if present in the river).

For benthic and hyporheic sediments, the main organisms concerned are decomposers (bacteria, fungi, micro and macro-invertebrates that feed on animal and plant debris), primary consumers (pelagic micro and macro-invertebrates).

The characterisation of exposure of the target organisms requires the evaluation of flows of CSOs and the river respectively to calculate the percentage of effluent in the river, and thus calculate the Predicted Environmental Concentration (PEC). It also requires establishing the range of dilution of the effluent to which the organisms will be exposed in the laboratory during the “effect evaluation” phase. It should be noted that each of these two flows varies considerably through the season and during rain events. In view to conserving the organisms of the river, we seek to assess the ecological risk for the most “critical” period, *i.e.*, that corresponding to CSOs discharged during the first few minutes of a storm, during a period when the river is in low flow regime.

#### 3.1.2. Priority Objectives

In the framework of this study, the priority objectives of the assessment were formulated in the following way:

- *the discharge of CSOs in the river must not disturb the pelagic organisms* living in the water column *given their ecological and societal importance (conservation of biodiversity, participation in the self-cleaning of the river, etc.)” [11,30,31,32],*
- *“nor must the discharge of CSOs in the river disturb benthic and hyporheic organisms* living in the river sediment and its subflow, *given their ecological importance” [10,33].*

**Figure 2:**
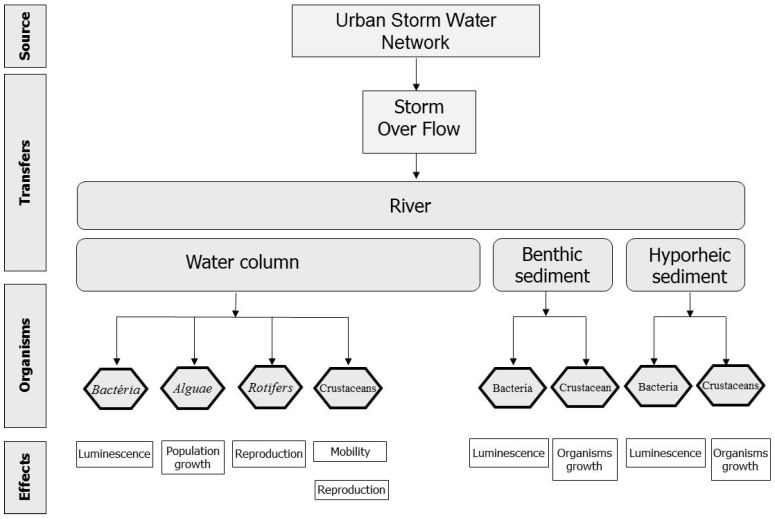
Conceptual model of the scenario studied.

#### 3.1.3. Formulation of the Conceptual Model

Figure 2 summarizes the different elements of the conceptual model, such as they result from the previous formulations. The organisms of the water column are represented by bacteria (*Vibrio fisheri),* single-celled algae (*Pseudo-kirchneriella subcapitata*), protozoa (*Brachionus calyciflorus)* and pelagic micro-crustaceans (*Daphnia magna* and *Ceriodaphnia dubia*). If fish are present in the river, a representative of these (*Brachydanio rerio*) is added to this battery (an option not represented in Figure 2).

The benthic and hyporheic organisms are represented by sedimentary bacteria and micro-crustaceans *(Vibrio fisheri* and *Heterocypris incongruens)*. Fewer bioassays are performed on the sediments than on the organisms of the water column in this initial version of the methodology. The two sedimentary organisms tested nonetheless have major advantages in favour of their selection in the methodology, that is to say they have shown their sensitivity to CSOs in the framework of previous studies, and they consume a little quantity of sample. This is a very important point from the practical viewpoint since the particles to be tested are produced by filtration of CSOs in the laboratory.

In this conceptual model, the parameters selected only concern a few organisms, and the representation of the resulting ecosystem is very simplified. It nonetheless corresponds to a good compromise between cost and the representativeness of the bioassays.

### 3.2. Materials and Methods

#### 3.2.1. Exposure Characterisation

##### General Approach

Exposure characterisation aims at determining the spatial-temporal contact between pollutants and target populations [19]. It includes the analysis of sources of pollutants and the distribution of pollutants in the environment. This analysis can be performed by using theoretical models of pollutant transfer and/or on the basis of experimental results. This phase concerns in the determination of one or more values characterising of exposure. In the case of a “substance-based” approach, the term Predicted Environmental Concentration (PEC) is used, whereas in a “matrix” approach, the notion of percentage of polluted source matrix in the environment is employed [16]. In both cases, the parameter concerned is the concentration that can be expected in the environment following different inputs.

##### Dilution of the Effluent in the River

In the framework of applying the methodology to a specific site in the future, the respective flows of the effluent and river during the “critical” period will directly depend on the configuration of the site in question. In the framework of the present task of formulating the methodology, these flows have been fixed by using existing data for a discharge site in the region of Lyon in France [34].

##### Sampling, Transport and Conservation of Percolate

The samples were taken manually from the discharge site in the region of Lyon mentioned above. The effluent was sampled during the first hour of heavy rain (about 80 L), then homogenised and divided into 2 L flasks for the different analyses and tests. All the measurements and experiments were performed as rapidly as possible following sampling. In the meantime the samples were kept in a cold room at 4 °C.

##### Physicochemical Analyses

The physicochemical analyses of the effluent were performed according to the protocols featuring in Table 1.

**Table 1:** Standards of the physicochemical analyses performed.

Parameters	Standards
pH	ISO 10523:2008
Conductivity	EN 27888
COD	NF EN ISO 10 304-1
Ammonium (NH_4_^+^)	NF EN ISO 11732
Phosphates (PO_4_^3−^)	NF EN 1189
Sulphates (SO_4_^2−^)	ISO 22743:2006
Chlorines (Cl^−^)	NF EN ISO 10 304-1
Heavy metals (Cd, Cr, Cu, Ni, Pb, Zn)	NF EN ISO 11885
PAH	NF EN ISO 17993

#### 3.2.2. Effect Characterisation

##### General Approach

This phase entails defining to what extent the organisms of the target ecosystem are significantly sensitive to the pollutants to which they are exposed [16]. This step is mainly based on biological approaches that include batteries of bioassays. A large number of batteries of bioassays have been proposed in the literature for different fields of study and matrixes. Mention can be made of those relating to: (1) substances [35,36,37]; (2) effluents [38,39,40,41]; (3) sediments [42]; (4) wastes [43,44,45,46]; and (5) soils, sludges and composts [47,48]. It relies on the acquisition of different values of ecotoxicological effects (NOEC, EC20, EC50, *etc*.) making it possible to calculate, after applying an extrapolation factor, and the value of absence of significant effect on the whole of the target ecosystem. This value is commonly known as the Predicted No Effect Concentration (PNEC). 

##### Matrixs Studied and Bioassays Performed

The effects on the organisms of the water column were characterised with CSOs filtered at 1.2 μm.

The effects on the benthic and hyporheic organisms were characterised with the particle fraction of CSOs, the fraction retained when filtering the CSOs at 1.2 μm. The protocols and parameters measured in each of the bioassays are given in Table 2. 

**Table 2:** Standards and parameters of the bioassays performed.

Organism	Standard/Protocol	Effect criterion (time of exposure)
*Daphnia magna* (Dm)	NF EN ISO 6341 (T 90-301)	Mobility (24 h and 48 h)
*Vibrio fischeri* (Vf)	ISO 11348-3	Luminescence (30 mn)
	Microtox® Basic Solid Phase Test	Luminescence (20 mn)
*Pseudokirchneriella subcapitata* (Ps)	NF T 90-375	Population growth (72 h)
*Brachionus calyciflorus* (Bc)	Protocol RoToxkit℘ F	Reproduction (48 h)
*Ceriodaphnia dubia* (Cdu)	NF T 90-376	Reproduction (7 j)
*Daphnia magna* (Dm)	ISO/FDIS 10706	Reproduction (21 j)
*Heterocypris incongruens* (Hi)	Protocol OstracodToxkit™ F	Organism growth (6 j)

Note: Grey background boxes refer to solid phase tests.

#### 3.2.3. Risk Characterisation

There is a large range of possible methods, of variable complexity, to carry out the stage of risk characterisation . The choice will depend on the operational constraints and the available data. Rivière notes “the ecological risk can be expressed in various manners: qualitative (absence or not of risk), semi-quantitative (weak, average and high risk), in probabilistic terms (the risk is *x*%)”. The method known as “the quotient” is the most widespread method for the semi-quantitative characterization of risks. This method consists in calculating the ratio (or quotient) which is expressed as a “Predicted Environmental Concentration” (PEC) divided by a “Predicted No Effect Concentration” (PNEC). When the quotient value “*Q*” is greater than 1, the risk is considered as significant, and becomes more extreme as the quotient increases. Conversely, the more the quotient falls below 1, the more the risk is regarded as low. This method was chosen for this methodology. The PNEC is estimated using the results of bioassays performed on the CSOs. To adjust the value of the extrapolation factor as a function of the nature and number of available test results, we refer to the recommendations of the Technical Guidance Document (TGD) drawn up by the European Chemicals Bureau which is responsible for classifying chemical substances [24].

## 4. Results

The approach described above was applied to CSOs of the discharge site in the Lyon region mentioned previously.

### 4.1. Exposure Characterisation

#### 4.1.1. Proportion of CSOs in the River

At the critical moment of the scenario, during the overflow of the first discharges of CSOs into the river, the flow of the effluent was evaluated at 1.0 L/s and the flow of the river was evaluated at 16 L/s for the studied site. These values lead the presence of 5.9% effluent in the river at the critical moment of the scenario. 

#### 4.1.2. Chemical Analyses

The results of physicochemical analyses of the CSOs filtered at 1.2 μm are given in Table 3.

**Table 3:** Physicochemical analyses of CSOs.

Parameters	Units	Concentration *
pH	-	8.8
Conductivity	µS/cm	967
DCO	mg/L	54
NH_4_^+^	mg/L	**17.3**
PO_4_^3−^	mg/L	8.9
SO_4_^2−^	mg/L	27
Cl^−^	mg/L	95
Cd	mg/L	<0.0001
Cr	mg/L	0.0018
Cu	mg/L	0.0037
Ni	mg/L	0.0011
Pb	mg/L	0.0004
Zn	mg/L	0.0100
Total hydrocarbons	mg/l	<0.1
Naphthalene	µg/L	<0.06
Acenaphthylene	µg/L	<0.02
Acenaphthene	µg/L	<0.02
Fluorine	µg/L	<0.02
Phenanthrene	µg/L	**0.03**
Anthracene	µg/L	<0.02
Benzo(a)anthracene	µg/L	<0.02
Dibenzo(ah)anthracene	µg/L	<0.02
Fluoranthene	µg/L	<0.02
Benzo(b)fluoranthene	µg/L	<0.02
Benzo(k)fluoranthene	µg/L	<0.02
Pyrene	µg/L	<0.02
Benzo(a)pyrene	µg/L	<0.02
Indeno(123-cd)pyrene	µg/L	<0.02
Chrysene	µg/L	<0.02
Benzo(ghi)perylene	µg/L	<0.02

* Single measurement

The pH of the effluent was slightly alkaline (8.8) and its conductivity was high, demonstrating the presence of a strong ionic charge. Also of note in the effluent was a non-negligible concentration of NH_4_^+^ (17.3 mg/L) and non-negligible concentrations of heavy metals (particularly copper and zinc). 

In contrast, the HAP concentrations are undetectable, except for the phenanthrene (0.03 µg/L) which nevertheless remains low. Globally, these results show pollutant concentrations which are in the range of what is generally observed in the CSOs.

The results of the physicochemical analyses practiced on the particle fraction of the CSOs are given in Table 4.

**Table 4:** Physicochemical analyses CSO particles.

Parameters	Units	Concentration *
**Heavy metals**		
Cd	mg/kg	0.7
Cr	mg/kg	24
Cu	mg/kg	420
Ni	mg/kg	34
Pb	mg/kg	92
Zn	mg/kg	1,190
**Loss on ignition (550 °C)**	g/kg	321
**Total hydrocarbons**	mg/kg	2,600
**PAH**		
Naphthalene	µg/kg	<50
Acenaphthylene	µg/kg	<50
Acenaphthene	µg/kg	<50
Fluorine	µg/kg	58.8
Phénanthrène	µg/kg	382.3
Anthracene	µg/kg	117.6
Benzo(a)anthracene	µg/kg	<50
Dibenzo(ah)anthracene	µg/kg	<50
Fluoranthene	µg/kg	235.3
Benzo(b)fluoranthene	µg/kg	<50
Benzo(k)fluoranthene	µg/kg	<50
Pyrene	µg/kg	205.9
Benzo(a)pyrene	µg/kg	<50
Indeno(123-cd)pyrene	µg/kg	<50
Chrysene	µg/kg	88.2
Benzo(ghi)perylene	µg/kg	<50

* Single measurement

The analyses show an important rate of organic matter in the particles (loss on ignition close to 32%), a high concentration in hydrocarbons, and a high concentration in PAH (especially for phenanthrene, anthracene, fluoranthene and pyrene much higher concentrations of heavy metals than in the filtered water) were also observed.

### 4.2. Effect Characterisation

#### 4.2.1. Bioassays on the Organisms of the Water Column

The results of the bioassays performed with CSOs on the organisms of the water column are presented in Table 5. The Efficient Concentration for 20% of the organisms (EC_20_) is always higher than 80 % of CSOs, which indicates a very low ecotoxicity of theses, for all the tested organisms.

**Table 5:** Results of bioassays on the organisms of the water column.

Organisms	Measured parameters	Results (% of CSOs)
*Vibrio Fischeri*	EC 20 (Luminescence)	>80
*Daphnia magna*	EC 20 (Mobility)	>80
*Pseudokirchneriella subcapitata* (Ps)	EC 20 (Growth)	>80
*Daphnia magna*	EC 20 Reproduction	>80
*Brachionus calyciflorus*	EC 20 Reproduction	>80
*Ceriodaphnia dubia*	EC 20 Reproduction	19.6
*Brachydanio rerio*	Test not performed due to the lack of fish in the studied little river	

#### 4.2.2. Bioassays on CSO Particles

The results of the bioassays performed on CSO particles are given in Table 6. The result of the first test (*Vibrio Fischeri )* indicates a strong ecotoxicity of the particles. Indeed, it is necessary to dilute fifty times the particles in order to enable the survival of the organisms.

The second test (*Heterocypris incongruens)* has been made only with undiluted particles. The ecotoxicity observed confirms the ecotoxicity of the solid phase of the CSOs.

**Table 6:** Results of bioassays on sedimentary organisms.

Organisms	Measured parameters	Results
*Vibrio fischeri* (on solid phase)	EC 20 (Luminescence)	0.2% of particles
*Heterocypris incongruens*	Mortality of organisms with undiluted particles	100% mortality with undiluted particles

#### 4.2.3. Risk Characterisation

The two objectives we chose in the current state of the approach are examined successively:

- *O1: “the discharge of CSOs in the river must never disturb the organisms of the water column”*

Risk can be characterised with the quotient method, *i.e.*, by comparing the PNEC (in % effluent) with the PEC, in this case equal to the proportion of effluent in the river downstream of the discharge (5.9%). To obtain the PNEC, an extrapolation factor is applied to it using experimental laboratory data whose value depends on the data considered [20,49]. In the case of our results, which focus on four chronic ecotoxicity assays and two acute ecotoxicity assays. The factor recommended by the Technical Guidance Document of the European Chemicals Bureau responsible for classifying chemical substances is a maximum of 10 with three chronic bioassays. So, we have chosen an extrapolation factor of 5. By applying this factor, we obtain a PNEC of 3.92% (19.6%/5). This leads to a risk quotient of 1.50 (5.9%/3.92%) which indicates a slight risk for the organisms of the water column.

- *O2: “the discharge of CSOs in the river must never disturb the benthic and hyporheic organisms”*

Here again risk can also be characterised by using the quotient method. To obtain the PNEC, we apply a higher extrapolation factor than the previous one; since the experiments concerned are carried out with a battery of fewer bioassays than before [20,49]. Under these conditions; and by taking into account the recommendations of the European Chemicals Bureau responsible for classifying chemical substances [24]; an extrapolation factor of 50 is proposed. By applying this factor to the results obtained for the particle fraction; we obtain a PNEC of 0.004% (0.2%/50).

Previous works [34] have shown that particles of CSOs can accumulate heavily at certain points of the river downstream of the overspill, notably in hyporheic sediments at downwelling zones. If, by way of example, we assume that this accumulation leads to the presence of 0.01 to 10% CSO particles in the sediment, depending on the sector of the river, this leads to a risk quotient between 2.5 (0.01%/0.004%) and 2,500 (10%/0.004%). These quotients indicate that CSOs present a very high risk for the organisms living in the sediment zones rich in CSO particles.

In brief, with the methodology formulated, the effluent presents a slight risk for the organisms of the water column downstream of the overflow, and a very high one for the sedimentary organisms, especially at downwelling zones in the hyporheic zone.

## 5. Discussion

### 5.1. The Results Obtained

The ecological risk estimated for the organisms of benthic and hyporheic sediments is far greater than that calculated for the organisms of the water column. On the one hand, this can be explained by the high concentration in particles estimated at certain points of these sedimentary compartments and, on the other hand, by the very high concentration of pollutants in these particles whose value far exceeds PNEC values for several parameters (Table 7) [50]. 

These conclusions, which emphasise the risk for sedimentary organisms, particularly in the downwelling zones of the river, are consistent with the observations made by other authors for analogous rivers [34,51,52,53].

Different management recommendations can be made on the basis of these assessments. Regarding the organisms of the water column, the calculated risk first depends on the exposure which itself depends on the concentration of pollutants and the ratio of the CSO and river flow rates. Since it is difficult to control the flow rate of the river, especially at low flow, three improvements for managing the effluent could be considered: (i) the reduction of pollutant emissions at source, (ii), the provisional retention of CSOs in a retention basin and then progressive discharge into the river following the “critical” period, and (iii) setting up a CSO treatment system. In our opinion, the most sustainable solution would be the optimised combination of these three solutions, with a gradual progression to solution 1. 

**Table 7:** Comparison of concentrations of pollutants with PNECs.

Pollutants	Concentration in particles		Sediment PNEC (INERIS, 2012)	Concentration in the filtered CSOs		Aquatic PNEC (INERIS, 2012)
Cu	mg/kg	420	0.8	µg/L	3.7	1.6
Cr	mg/kg	24	-	µg/L	1.8	4.1
Pb	mg/kg	92	6.8	µg/L	0.4	5
Zn	mg/kg	1,190	26	µg/L	10	8.6
Phenanthrene	µg/kg	382.3	2.3	µg/L	0.03	1.34
Anthracene	µg/kg	117.6	31.2	µg/L	<0.02	0.063
Fluoranthène	µg/kg	235.3	2.3	µg/L	<0.02	0.1
Pyrene	µg/kg	205.9	23	µg/L	<0.02	0.012

Regarding the particle fraction of the CSOs, and the component most responsible for risks to the river, the main, and also inexpensive, improvement for management considered is to install a settling pond downstream of the network intended to collect the CSO particles before their discharge into the river. 

### 5.2. The Methodology Formulated

It is clear that the representation of the aquatic ecosystem as defined in the formulation of the conceptual model is greatly simplified, particularly for the assessment of effects on sedimentary organisms. To evaluate these effects, some authors have proposed batteries of bioassays that go into much greater depth and which are adapted to each situation [54], or working with procedures more representative of ecosystems such as microcosms and mesocosms [43,55]. These approaches are nonetheless far more costly and are not always “economically acceptable” when implemented in the field. Lastly, the real issue is to know whether the battery of bioassays selected permits adequate protection of the ecosystems concerned. 

On the contrary, the methodology proposed provides a real improvement in comparison to usual practices in the area of risk assessment [16] as account is taken of the impact on sedimentary organisms, especially those of the hyporheic zone. 

Besides, given the importance of fixed epibenthic organisms (periphyton) in the general ecological functioning of the river, further work is necessary to study the pertinence of including them in the assessment of impacts. 

Since CSOs are effluents loaded with heavy metals, the tool formulated should be adapted to take better account of bioaccumulation in the river. This could be done by using the periphyton as a bioaccumulation measurement tool, something that is often done for the *in situ* monitoring of rivers [56,57,58,59].

CSOs can also contain pollutants liable to lead to genotoxic effects. Although it is still unclear as to the additional risk this type of effect can generate, its potential consequences are such that they cannot be neglected, thus they should be considered in risk assessment procedures for ecosystems [60,61,62,63]. The advantage of studying the integrity of DNA as a biomarker of genotoxicity in the framework of risk assessments, especially in the long-term, resides in the persistence of genotoxic response both for the organism and its offspring [60,64,65,66].

The quotient method chosen to calculate and express risks is quick and provides an efficient tool for communicating the results. However, it is a relatively succinct means of characterising risks and is based on a certain number of simplifications: (i) effects and exposure are both simplified into a value, which may hide conceptual biases, for example, the fluctuation of exposure concentration; (ii) indirect effects, for example eutrophication, are not easily taken into account. Other risk characterisation methods can be used in certain contexts [19,67,68]: (i) qualitative methods that characterise risk into two or three categories, for example, high/low/medium, most often on the basis of expert judgement [20]. They can be used for comparative approaches (with, for example, two types of contamination), (ii) methods incorporating the global pollutant/response to estimate the level of risk linked to a given exposure level. These methods are particularly useful for testing several possibilities of risk reduction, or when different exposure concentrations (as a function of time and geographical area) and or effect concentrations (chronic/acute) exist [69,70].

## 6. Conclusions

This study showed that it is possible to assess risks for the different compartments of a river generated by the discharge of CSOs, using relatively accessible and standardised ecotoxicological investigation resources. Other ecological risk assessment approaches exist around the world. The purpose of our approach, other than taking into account the characteristics of the host environment, and specifying the impact of the effluent on its different compartments, is to define the compartments impacted and improve the realism of the assessment, in view to optimising management decisions. In this respect it is similar to the “Waste ecocompatibility” assessment approach established in France by the Environment and Energy Management Agency to assess the impact on ecosystems of storage scenarios and the reutilisation of mineral wastes for construction [27].

For all that, the methodology presented can, and must, be improved still further in several directions so it can be used operationally by organisations responsible for managing rivers. It is advisable to check whether the battery of bioassays selected for the sedimentary organisms should be completed, and whether organisms representing fixed epibenthic organisms should be included. When considering these points, it should not be forgotten that a limited number of biological responses leads to increasing the uncertainty on the assessment of effects, and thus increasing the value of the final risk quotient through the necessary augmentation of applicable extrapolation factors.

Lastly, works aimed at improving the final phase of risk characterisation, which is currently based solely on the quotient method, and at ensuring the clarity of the results on the associated uncertainty are required to permit optimal use of the tool formulated [16,67,68].
